# Circulating Tumor Cells Measurements in Hepatocellular Carcinoma

**DOI:** 10.1155/2012/684802

**Published:** 2012-05-28

**Authors:** Franck Chiappini

**Affiliations:** INSERM U785, AP-HP Hôpital Paul Brousse, Centre Hépato-Biliaire, Université Paris-Sud. 14 avenue Paul Vaillant-Couturier, 94800 Villejuif, France

## Abstract

Liver cancer is the fifth most common cancer in men and the seventh in women. During the past 20 years, the incidence of HCC has tripled while the 5-year survival rate has remained below 12%. The presence of circulating tumor cells (CTC) reflects the aggressiveness nature of a tumor. Many attempts have been made to develop assays that reliably detect and enumerate the CTC during the development of the HCC. In this case, the challenges are (1) there are few markers specific to the HCC (tumor cells versus nontumor cells) and (2) they can be used to quantify the number of CTC in the bloodstream. Another technical challenge consists of finding few CTC mixed with million leukocytes and billion erythrocytes. CTC detection and identification can be used to estimate prognosis and may serve as an early marker to assess antitumor activity of treatment. CTC can also be used to predict progression-free survival and overall survival. CTC are an interesting source of biological information in order to understand dissemination, drug resistance, and treatment-induced cell death. Our aim is to review and analyze the different new methods existing to detect, enumerate, and characterize the CTC in the peripheral circulation of patients with HCC.

## 1. Introduction

Hepatocellular carcinoma (HCC) is responsible for significant morbidity and mortality in cirrhosis and also accounts for between 85% and 90% of primary liver cancer [1–3]. Most of HCCs in the world occur in the setting of cirrhosis and over half-million of people develop liver cancer every year and an almost equal number die of it [1, 2, 4].

The most important causes leading to HCC are the HBV and HCV infections, heavy alcohol consumption, aflatoxin B1, age and gender (males are more susceptible than females), race (Asian and African over 20 years old), tobacco consumption, obesity associated with nonalcoholic fatty liver disease, and the increase of the Diabetes II mellitus (that rises the risk factor between 2 and 3), genetic hemochromatosis, primary biliary cirrhosis, and alpha1-antitrypsin deficiency and autoimmune hepatitis [1, 2, 5–32].

Usually, HCC develops during a long process of inflammation and fibrosis, eventually leading to cirrhosis [2, 16, 33, 34].

HCC is one of the most aggressive cancers. Patients who show progress over the terminal stage have 1-year survival of less than 10%. The choice of the therapy and the prognosis are dictated by the severity of the liver function, portal hypertension, and medical comorbidities. National and international consensus was established to choose the best treatment adapted for each case and obtain the best prognosis [1, 5–21, 24, 25, 27, 30, 35, 36].

In the field of biology of tumors, some expressions have been coined for the different types of circulating cellular elements. The term *circulating tumor cells* (CTC) defines specifically the tumor cells detected in blood or lymphatic vessels. Circulating cells in the bloodstream or in the lymphatic system are considered to be tumoral microemboli (CTM) and represent a collective migration. The terms disseminated tumor cells (DTCs) and isolated tumor cell (ITC) can be also found in the literature but are usually used to define the cells that can be detected in both the organs and the bloodstream. The word micrometastasis is usually used to indicate tumor cells found in distant organs, the tumor spread circulating of liver-derived cells [20, 37, 38].

The presence of CTC reflects the aggressiveness nature of a solid tumor. Many attempts have been made to develop assays that reliably detect and enumerate these cells. The clinical results obtained with such assays suggest that in some tumor types, CTC detection and identification can be used to estimate prognosis and may serve as an early marker to assess antitumor activity of treatment. In addition, CTC can be used to predict progression-free survival and overall survival. CTC are an interesting source of biological information in order to understand dissemination, drug resistance and treatment-induced cell death [22, 23, 39–50].

In HCC animal models showed that 10 to 10 000 CTC are capable to initiate new metastasis [20, 51–53]. Even after curative resection, the tumor recurrence rate remains high. Although CTC detection has been applied and well documented in different types of cancer, especially breast cancer, CTC detection is not routinely performed in HCC follow-up and remains in the experimental field. However, CTC detection might bring new interesting information of metastatic process might be used as diagnostic tool of early recurrence and may allow a better patient selection for liver transplantation. Mechanisms of tumor recurrence are still poorly understood. Several arguments point out that HCC tumor cells can infiltrate the blood system as shown by the presence of alpha-fetoprotein mRNA [54–64]. CTC seem to be correlated with poor survival in many types of tumors [54, 55, 57, 58, 62, 64].

However, HCC circulating cells are still difficult to detect, and their presence and amount are poorly correlated with either long-term survival or recurrence in the setting of HCC.

Methods of CTC detection have to be highly sensitive and specific. The first technical challenge in this field consists of finding exceptional cells. Just a few CTC are mixed with the approximately 10 million leukocytes and 5 billion erythrocytes in 1 mL of blood. To be useful, the method used to identify circulating tumor cells must also detect all tumor cells and discriminate them from nontumor cells. [22, 37, 39, 40, 43, 47, 48, 65–67]. It is important first to have circulating hepatoma-specific biomarkers to be able to detect the CTC and further to be useful to early diagnosis, monitoring metastasis, or posttreatment recurrences of HCC [68].

In this paper, we will first focus on the specific markers of HCC used to detect CTC, follow by the different methods of enrichment of CTC and finally we will describe the new promising methods to study and detect the CTC from the HCC.

## 2. HCC Markers and Their Applications for CTC

The hepatocellular carcinomas can synthesize various tumor-related proteins, polypeptides, and isoenzymes more or less specific of the hepatoma tissues as well as the corresponding mRNA. It is important to have tumor-specific markers of HCC to detect CTC in the bloodstream or lymphatic system.

### 2.1. Relevance of Cytokines in HCC Circulating Tumor Cells

HCC tumor has shown to secret a lot of cytokines related to the development of the tumor, like vascular endothelial growth factor (VEGF), transforming growth factor-beta 1 (TGF-*β*1), Interleukin 8 (IL-8), or tumor-specific growth factor (TSGF). These serum markers are useful to follow-up the development and the prognosis of the HCC but useless to follow-up circulating tumor cells in blood [69]. Because of that the detection of the cytokine(s) and the mRNA marker(s) simultaneously increases the specificity and the sensitivity the methods of detecting CTC.

### 2.2. *α*-Fetoprotein (AFP)

The serum *α*-fetoprotein (AFP) levels show high levels in newborns and then decline progressively below 10 ng/mL in 300 days of life. An increase of serum AFP levels can be observed during pregnancy, and in patients with mucoviscidosis, acute hepatitis (30%–50%), chronic hepatitis (15%–50%), cirrhosis (11%–47%) and other cancers (gastrointestinal, pancreatic, biliary, nonseminomatous germ-cell testicular, and germ cell ovarian). Serum level AFP sensitivity is between 39% to 64% with approximately 60% for a cutoff of 20 ng/mL and decrease to 22% if higher cutoff of 200 ng/mL is used [9, 70–74]. AFP specificity is around 76%–91% with a low predictive value between 9%–32% [9, 70–72].

In addition, the serum AFP level is correlated with the tumor size. 80% of small HCC (<2 cm) do not express AFP. In the other hand, AFP level can be elevated in patients with chronic liver disease with high degree of hepatocytes regeneration such as HCV-infection that show a high level of AFP in absence of malignancy [75, 76]. For these reasons, some additional serological markers used in combination with AFP seem to improve the performance of this biomarker alone, especially in term of sensitivity.

Studies were done by using one or two more markers like des-*γ* carboxyprothrombin (DCP) also called prothrombin induced by vitamin K absence II (PIVKAII) and glycosylated AFP-L3 (*Lens culinaris *Agglutinin-Reactive AFP) fraction serum levels to diagnose earlier HCC and increasing the sensitivity especially when HCC is associate with cirrhosis, HCV or HBV infection [71, 74, 77–81]. But there is no study that correlates their serum levels and the circulating tumor cell during the HCC development.

Because of that serum AFP and mRNA AFP detection can be used.

The relevance of AFP mRNA as a marker of circulating tumor cells is better but is also controversial because these cells have not been further characterized, and it has been shown that they may correspond to normal circulating hepatocytes [58, 82, 83]. Furthermore, these tumor cells have mostly been sought and detected shortly after liver resection [56, 58, 62, 82, 83]. This finding suggests that CTC could spread following liver mobilization or manipulation. Although the mechanisms leading to intra, and extrahepatic recurrences are still unknown, some observations suggest that bone marrow (BM) could also be a specific reservoir of CTC. Indeed, several reports have suggested that tumor cells are from BM origin [84–86]. Hepatic tumor stem cells may take advantage of the potential for stem cell support of the BM microenvironment. The amplification of AFP mRNA by means of reverse transcription (RT) and a nested polymerase chain reaction (PCR) is the highly sensitive method for the detection of residual HCC cells in peripheral blood. The qualitative (positive versus negative) detection of HCC circulating tumor cells in blood samples from individual patients is of limited value in predicting the risk of disease progression. Because the level of AFP mRNA is increased in HCC tissue compared with in normal hepatocytes, the quantification of AFP transcripts seems to be a more reliable indicator of disease progression. A more highly sensitive assay based on TaqMan technology to quantify AFP mRNA in “real time” should be preferred [59, 87–89]. Even using this methodology, reported results are not homogeneous and contradictory [72]. The main studies which have evaluated AFP mRNA are summarized in Table 1. The false-positive results can be obtained using AFP mRNA.

### 2.3. Alpha Albumin (ALF)

For more than a decade, we know that mRNAs of hepatocytes-specific albumin genes are detected in peripheral blood by RT-PCR. It was shown that there is evidence that detection of albumin mRNA associated with the detection of AFP mRNA is strongly associated with the presence of metastases [57, 61, 94, 95]. Wong et al., showed that circulating hepatocellular carcinoma cells can be detected and be semiquantified by albumin RT-PCR [96]. On the other hand, Resto et al., showed that downregulation of alpha-albumin (ALF) specifically in HCC-circulating cells can be used as a specific marker to discriminate the normal hepatocellular circulating cells that express abundantly ALF. RT-PCR ALF in association with RT-PCR AFP have been proposed to distinguish normal or malignant hepatocytes in peripheral blood, but the interpretation of the results is still debated [97].

### 2.4. Transforming Growth Factor Beta-1 (TGF-*β*1)

The levels of circulating TGF-*β*1 and TGF-*β*1 mRNA were significantly higher in the HCC patients than any other group of patients. The sensitivity and specificity of circulating TGF-*β*1 level (>1.2 *μ*g/L) were 90% and 94% for HCC diagnosis, but no significant correlation was found between TGF-*β*1 expression an AFP levels or tumor size. The combined detection of TGF-*β*1 and serum AFP could raise the detection rate of HCC up to 97%. Both of circulating TGF-*β*1 and TGF-*β*1 mRNA could be used as sensitive biomarkers for diagnosis and prognosis of HBV-induced HCC [98, 99]. Unfortunately, TGF-*β*1 mRNA was poorly studied and further investigations have to be done to use circulating TGF-*β*1 mRNA as a marker of circulating tumor cells in HCC.

### 2.5. Insulin-Like Growth Factor (IGF)-II

Studies using amplified fragments of IGF-II mRNA by RT-PCR showed that the lowest sensitivity with 2 ng/L of total RNA. Dong et al., showed that the positive frequencies of IGF-II mRNA were 100% in HCC, around 50% in paracancerous and 0% in noncancerous tissues respectively. But, the positive frequency of circulating IGF-II mRNA was 34% in HCC, and no amplification was found in other liver diseases, extrahepatic tumors, and normal control, meaning that IGF-II is specific of the HCC but not really sensitive. Associated to other circulating markers IGF-II can be helpful to detect CTC. The circulating IGF-II mRNA was correlated with the stage of HCC (incidence = 100%) with extrahepatic metastasis and 35% with AFP-negative. No difference was found between tumor size and circulating IGF-II mRNA [99, 100] but these results are controversial [101].

### 2.6. Prostate-Specific Antigen (PSA)

The Prostate-Specific Antigen (PSA) had shown to be well-established reliability marker and remained a valid prostate marker in patients with acute hepatitis and HCC [102]. But these results are controversial, PSA and mRNA PSA seem to do not be specific to the tissue and frequently detected in peripheral blood cells from healthy patients [17]. In addition, like the cytokines, serum PSA cannot be used as hepatocellular carcinoma marker for circulating tumor cells.

### 2.7. Heat Shock Protein (HSP)

Heat shock proteins (HSPs) are stimulated under perturbation or stressors by the tissue. HSPs are ubiquitous molecules and can be also expressed during carcinogenesis. Different HSPs have been related to the development of the hepatocellular carcinoma like gp96 or GRP94, HSP70 and HSP27, but none of them were used as a specific marker of circulating tumor cell [99].

### 2.8. Human Telomerase Reverse Transcriptase (hTERT)

Human telomerase is a ribonucleic protein composed by the association of three structures: human telomerase RNA component (hTERC); human telomerase-associated protein 1 (hTEP1); human telomerase reverse transcriptase (hTERT). hTERT is the catalytic unit of the complex. Also, telomerase is expressed in embryonic cells, in most human cancer cells or immortal cell lines, but not in normal somatic cell lines or tissues. For these reasons hTERT was investigated as a marker of diagnosis and prognosis of HCC, but the results are controversial and appear that false-positive results can be observed because of lymphocytes, precancerous liver parenchymal cells, and micrometastasis maybe responsible (for review [69, 99, 103]). Recently, Kong et al. investigated hTERT in peripheral blood in HCC from 343 Korean patients. There is no association between hTERT expression and clinical features and not a relationship between AFP and hTERT mRNA. Their conclusion is that AFP and hTERT mRNA expression in peripheral blood is useless as HCC prognostic markers [104].

### 2.9. Cancer-Testis Antigens (CTA)

Cancer-testis antigens (CTAs) represent a category of tumor-associated antigens normally expressed in male germ cells but not in adult somatic tissues [105]. CTAs are heterogeneous group of antigens. Actually, more than 44 distinct CT “gene” or “antigen” families have been reported in literature. Certain CT gene families contain multiple members, as well as splice variants and today more than 89 distinct transcripts are known to be encoded by CT genes [105]. A number of CT antigens have been found expressed with high percentage and specificity in HCC. The expression of the CT antigens mRNA was investigated by Wu et al., in HCC and corresponding peripheral blood of 37 patients with HCC, 15 samples of cirrhotic tissues and 15 normal tissues with the same method. Two CT antigens SSX-2 and SSX-5 showed in this study high specific and high frequent expression only in HCC tissues. In corresponding peripheral blood of HCC tissues, the positive expressions rate of these two CT antigens mRNA was not very high [106]. The same group of researchers used another two CT antigens SSX-1 and NY-ESO1 in the same group of patients and with the same methods (RT-PCR) with the corresponding peripheral blood. They showed that SSX-1 can be potential used in peripheral blood, with short-term recurrence rate at 46% (6/13) in patients whose peripheral blood expressed SSX-1 mRNA, while the recurrence rate in patients with negative SSX-1 mRNA was 28.6% (4/14) [107]. In another study, Peng et al., showed that specific expression of CT antigens was observed in AFP-negative HCC, suggesting the application of their mRNA as tumor markers to detect circulating HCC cells [108]. Yang et al., showed that FATE/BJ-HCC-2 (another CTA) mRNA expression was detected in the peripheral blood mononuclear cells (PBMCs) of 46.67% patients, whose HCC tissue samples were cut off and positive for FATE/BJ-HCC-2 mRNA, which implicated tumor cell dissemination in blood circulation and related to the metastasis of HCC. These studies suggest that CT antigens expressions can be used in peripheral blood to detect HCC circulating cells but also can be associated to the research of AFP mRNA to increases the specificity and the frequency of the method. This group of markers seems promising and further studies have to be done first to determine the panel of CT antigens to be used as markers of HCC circulating cells.

## 3. New Approaches to Discover New Markers

To attempt the lack of CTC markers, new techniques and technologies are used such as microarray/mRNA large analyses, proteomic and “secretome” analyses and finally microRNA testing.

### 3.1. Microarray/mRNA Large Analyses

DNA chips were used to measure and find new markers to diagnose HCC but also to use these as CTC markers. The studies showed the expression of mRNAs for members of the glypican and syndecan families of heparin sulfate proteoglycans such as GPC3. GPC3 can be a good CTC marker that can be used in human or mouse models [109, 110]. Another interesting marker was discovered called Snail. Snail mRNA was studied in blood of patients with HCC and metastasis [80]. But further investigation has to be done to figure out the specificity and the sensitivity of those markers.

### 3.2. Proteomic and Secretome Analyzes

In the process to find new markers for CTC, a number of teams started to work with proteomic analysis such as quadrupole IT-TOF, SELDI- TOF MALDI-TOF/TOF mass spectrometry. Their objective is tracking earlier the development and the progression of the HCC. Few markers or group of markers were identified by these methods such as the usual markers AFP, AFP-L3, TGF-*β*1, and PIVKA-II but also vitronectin, alpha-1-fucosidase (AFU) and DCP, Golgi protein-73 (GP73), hepatocyte growth factor (HGF), and nervous growth factor (NGF) [111–117].

Interestingly, the proteomic analyses were able to detect new markers in the serum secreted (which is called “secretome”) by the carcinoma cells. [118–120]. Over 90 proteins in some studies compiled with high powerful biocomputational analysis where identified and used to diagnose HCC early [111, 113, 115, 116]. Unfortunately these studies did not analyze the real usefulness of these markers to identify CTC in the patients with HCC.

A subgroup of HCC was identified by these techniques expressing stem cell markers (CD133, CD90, CD44, EpCAM, CD13 or neural cell adhesion molecule; NCAM) defining what is called now liver cancer stem cell, but unfortunately these markers were not study in the area of circulating tumor cells [121–123], but they are very promising markers. Another group of markers very promising to detect CTC belong to the chemokine receptors such as CXCR4, CX3CR1, and CCR6 express during HCC progression [123, 124], but none of them were tested during a clinical trial.

### 3.3. MicroRNA Markers: A New Hope

Around 28 years ago, microRNA (miRNA) was discovered and showed the regulation of genes [125, 126] such as oncogenes or tumor suppressor genes at the level of the mRNA. More than 35 studies focused on the identification of miRNA or a group of miRNAs to be used as marker of early diagnosis or metastasis. miR-122/-122a, miR-221/222, miR-145, miR-146a, miR-26 (NF*κ*B pathway), miR-199a-3p (mTOR pathway), and miR-26 (MYC pathway) were strongly linked to the development and metastasis of the HCC and also a group of miRNAs were used to identify and classify HCC [127–132], but none of them were used as a CTC marker and tested during a clinical trial.

### 3.4. Cancer Stem Cell (CSC) Marker in HCC Associated with Flow Cytometry

Fan et al. described a multicolor flow cytometry used to detect the number of circulating CSCs (CD45− CD90+ CD44+) in the peripheral circulation HCC patients. Circulating CSCs predicted posthepatectomy HCC recurrence with high accuracy. They may be the target of eradication in the prevention of posthepatectomy HCC metastasis, recurrence and also will be used to monitor the efficiency of postsurgery treatment of the HCC [133]. The major problem of this technique beside the cost of the equipment is the fact that it detects only a part (cancer stem cells) and not the whole CTC decreasing the sensitivity of the method and increasing false-negative results.

## 4. Conclusion

In conclusion, we can observe that not too many specific HCC markers are available and useful for the detection of the CTC. This is certainly due to the heterogeneity of the hepatocellular carcinoma. The most important marker used in clinical routine is the detection of serum AFP mRNA expression (Table 1). But this marker is not expressed in all HCC and by consequence in all CTC leading false-negative results. Some propose to combine the research of more than one marker to increase the specificity and the sensitivity of CTC detection method. One of promising marker is Cancer-Testis Antigens, but more studies need to be done to select one or more CTA combined (or not) with the detection of the AFP mRNA expression. As we notice previously, CTC are very rare in peripheral blood. We saw also that real-time polymerase chain reaction is a method that in addition to be specific by the nature of the primers used it can amplify the signal by increasing the number of copies of mRNA originally presents in the sample.

Interestingly, the Milan Criteria and Model for End-Stage Liver Disease (MELD) system for the allocation of livers in the United States do not include CTC markers to select the recipient [134]. Among all of these CTC markers described above, only AFP is routinely available before transplant. Although its performance as a screening test is unsatisfactory [135], but it is a valuable predictor of HCC recurrence and patients survival after transplant [136, 137]. In Korea, a scoring system incorporating tumor size, number, and AFP has been proposed for the selection of transplant candidates [138]. These data suggest that serum AFP could be integrated with the Milan Criteria and the MELD system's HCC to improve selection criteria for prioritization patients on the waiting list. Following this idea, it will be interesting to assess the consequences of including more than one CTC markers in the scoring system to select HCC patients for liver transplantation and their followup.

But before using these markers, it is necessary to concentrate the number of CTC from the peripheral blood in a smaller volume. The next paragraph will describe these methods.

## 5. Enrichment of the Sample

CTC are usually detected in the peripheral circulation, but we can find CTC in other body fluids like the cerebrospinal fluids or the urines. The limitations to discover the CTC in these fluids are the same than in the blood circulation. However, it is possible to extract a relatively big amount of blood without harming the patient and much easier.

We will focus on the methods of CTC detection in the blood. As we describe above, CTC in the peripheral circulation occur at an estimated number of one CTC per 10^5-7^ peripheral blood mononuclear cell or PBMC. Because of the scarcity of the target cells, it is necessary to concentrate the sample. Since enrichment will inevitably be accompanied by some loss of CTC, irrespective of the method, some essays are performed directly in whole blood [89].

Two different groups of techniques can be used to enrich samples, the nonspecific and the specific enrichment techniques. The nonspecific enrichment techniques use physicochemical CTC properties (size, density, etc.). The specific enrichment technique uses markers expressed by the CTC. Advantages of the nonspecific and specific enrichment techniques are summarized in the Table 2 and described in the following paragraphs.

## 6. The Nonspecific Enrichment Techniques

### 6.1. Density Gradient(s) Centrifugation

The tumor cells, epithelial cells, platelets, and low-density leukocytes from leukocytes and erythrocytes can be separated by the propriety of their particular density (Table 2). Density gradient centrifugation is the preferred method to purify cells, subcellular organelles, and macromolecules. Density gradients can be generated by placing layer after layer of gradient media such as sucrose in a tube with the heaviest layer at the bottom and the lightest at the top in either a discontinuous or continuous mode. The cell fraction to be separated is placed on top of the layer and centrifuged. Density gradient separation can be classified into two categories: (1) Rate-zonal (size) separation. (2) Isopycnic (density) separation.

Rate-zonal separation takes advantage of particle size and mass instead of particle density for sedimentation [157].

In isopycnic separation, a particle of a particular density will sink during centrifugation until a position is reached where the density of the surrounding solution is exactly the same as the density of the particle. Once this quasi-equilibrium is reached, the length of centrifugation does not have any influence on the migration of the particle. A common example for this method is separation of nucleic acids in a CsCl gradient. A variety of gradient media can be used for isopycnic separations [157–161].

In the context of the CTC enrichment by centrifugation the isopycnic separation is the method usually used. The cells that have a density higher than the density of the buffer will stay in the bottom of the tube. If the density of the cells is lower than the buffer, they will remain on the top of the liquid, forming a ring. On the contrary, if the density of the cells is the same than the buffer, the cells will form a ring in the middle of the tube. A well-known example of the method is the commercial buffer Ficoll tube or Ficoll-Paque PLUS (GE Healthcare Bioscience, Amersham Biosciences AB) or Lymphoprep (Nicomed) to separate the red blood cells from the other cells including CTC (Table 2).

OncoQuick (Greiner) method uses a specific buffer able to isolate the CTC [47, 139, 140]. These methods are usually fast but expensive and found in a context of clinical laboratory used in routine diagnosis (Table 2). Alternative and cheaper methods can be used by preparing in the laboratory the gradient/density buffers. The same tube can contain one, two or three gradient buffers to increase the specificity of the separation between the different cells present in the blood [27, 47, 162, 163].

### 6.2. Lysis Buffers

The content of different cells has different osmotic pressures. It is possible to expose the samples to buffer(s) that can be hypo-or hyperosmotic to any cell different to the target cells. After the lysis step the mix is centrifuged and the pellets will contain the CTC. Some companies provide a kit with lysis buffers ready to use. After lysis, the next step is the extraction of DNA or RNA (e.g., Red Lysis Buffer from Qiagen or Panomics) or the extracted cells can be purified by immunomagnetic beads enrichment [147–149]. However, methods using lysis buffer can induce the death of a lot of cells including the CTC and it is not appropriate if the sample contains few CTC leading to false negative results.

### 6.3. Cytocentrifugation (Cytospin)

The cytocentrifugation was designed for hypocellular fluids; it spins at lower speeds and has more gradual acceleration and deceleration than normal centrifuges. Some are able to deposit cells directly onto a slide for examination. Cytocentrifugation could be used in research purposes and is also widely used in the routine surgical pathology practice. This method is fast and affordable [65, 144, 145, 164].

Methods to identify CTC can after be used (see below). As it occurs with magnetic beads, cytospin increases mortality of the target cells (Table 2). Because enrichment by cytocentrifugation is a critical step, addition of 10% buffered formaldehyde solution added to the blood sample can preserve morphology of the cells and will certainly preserve nucleic acids integrity [143], but the disadvantage of this method is that formaldehyde kills the cells (Table 2). Liquid-based cytology (LBC) using a filtration process and computer assisted thin layer deposition of cells has been developed as a replacement for cytocentrifugation and/or smearing, owing to its improved cell recovery capabilities and better cell preservation. In most published series, LBC allows a good interobserver reproducibility [165–167].

There are several advantages to this system. One is that it produces a thin layer of cells which is easier to evaluate than a thick smear. The morphology of the cells is also better. In addition, the entire cell sample is captured in the fixative vial which leads to a more representative smear being prepared. One of the most important advantages of this test is that the material that is left over after a smear has been prepared can be used for adjunctive testing. A further advantage is that the smears may be initially subjected to image analysis. Computer software “reads” the smears and registers the coordinates of the fields with what it regards as the most abnormal cells. On review, the system directs the cytotechnologists to these fields where they are evaluated. This can cut down on technologists' screening time (Table 2).

There are also some disadvantages, which include increased manpower needed to prepare the smears and the dependence of smear preparation on the instrument. This technique cannot be used directly from blood samples. The red blood cells need to be eliminated (See Table 2) and after the sample can be processed by this technique (Table 2). However, optimization of cell capture and fixation can be achieved by methods other than Cytyc Thinprep LBC, particularly while using meticulous modern cytocentrifugation methods in the study of hypocellular fluids [166, 167]. In their study, Piaton et al. conclude that Cytyc Thinprep LBC and modern cytocentrifugation techniques are appropriate methods for cytology-based molecular studies. From an economical point of view (standard cytocentrifugation are around $ 538 compared to Cytyc ThinprepH $ 1,278), and taking into account the value of a meticulous technique, cytocentrifugation with disposable sample chambers remains the quality standard for current treatment of urinary samples for example [166].

### 6.4. Filters

A nonspecific method of enrichment using filters can captured the cells with a certain size. The cells captured on the filter can after be transferred and analyzed on a slide. In this case the samples can come from blood or body fluids (urines, cerebrospinal fluid, and ascites). We will describe tow kinds of methods using this technology and usually used to isolate CTC (Table 2): the Isolation by Size of Epithelial Tumor Cells (ISET) method and the Micro-Electro-Mechanical System (MEMS).

#### 6.4.1. The Isolation by Size of Epithelial Tumor Cells

(ISET) method (Metagenex, Paris, France, http://www.metagenex.fr/) separates cells by size with a filter. Cells larger than 10 *μ*m, including tumor cells from carcinomas, are enriched from leukocytes (erythrocytes are lysed, see above) on a filter. Enriched cells are stained on the filter and CTC are precisely counted after cytopathological evaluation. The cells on the filter can be also studied by immunolabelling, FISH, TUNEL, and molecular analysis. Molecular analysis can be performed specifically to CTC after laser microdissection. The filter can be also mounted between slide and coverslip for routine microscope observation and storage. Although promising, this method is expensive, time consuming, and the filters are not easily available [47, 68, 141]. Cells should be better characterized using morphological methods that allow both detection and characterization. A second potential main advantage is that CTC could be compared to the primary tumor in order to better understand the mechanism of metastatic process. However, this approach has been rarely performed and neither firm recommendation nor conclusion could be drawn. The technique also avoids damaging the tumor cell which can be diagnosed using a simple pathologist analysis. However, the pathologist should get use to this technique to avoid a misinterpretation with others types of cells. The use of ISET technology to detect and characterize CTC in HCC has been reported in one study. Vona et al. reported that microemboli and isolated CTC could be detected in HCC patient [142]. Presence of CTC was associated with a shorter survival. This work also showed the feasibility of molecular studies of individual circulating cells. Indeed, *β*-Catenin mutations were searched in samples of 60 single microdissected CTC. *β*-Catenin mutations were found in only 3 CTC that highlighted the weak impact of these mutations in the initial step of tumor cell invasion. Further studies are needed.

#### 6.4.2. The Micro-Electro-Mechanical System (MEMS)

It is a parylene membrane microfilter device for single stage capture and electrolysis of circulating tumor cells in human blood and the potential of this device to allow genomic analysis. After the CTC, are captured in the filter, electrical lysis of cells on membrane filter is applied and the DNA as well as RNA can be extracted and analyzed by PCR or RT-PCR, respectively. CTC enrichment is performed by either gradient centrifugation of CTC based on their buoyant density or magnetic separation of epithelial CTC, both of which are laborious procedures with variable efficiency, and CTC identification is typically done by trained pathologists through visual observation of stained cytokeratin-positive epithelial CTC. These processes may take hours, if not days. The Micro-Electro-Mechanical System (MEMS-) based makes the process simpler faster and better to separate CTC (~90% recovery) from blood cells. Since enrichment will inevitably be accompanied by loss of CTC, irrespective of the exact method, some essays are performed directly in whole blood [89]. But the disadvantages of this technique are that morphology of the cells is lost, besides markers also and the capacity to count exactly the number of CTC (Table 2).

## 7. The Specific Enrichment Techniques

The specific enrichment techniques can use specifically protein tumoral markers expressed by the CTC. These methods use antibodies against the protein tumoral markers coupled to steel beads, by applying a magnetic field the cells expressing the marker can be captured. Several immune-magnetic methods (MACS system, Deanabeads Invitrogen, macroiron beads, ferrofluid(colloidal iron-) based systems) to enrich the sample have been used successfully [168]. Another approach to enrich the sample is to use the properties of CTC to grow-up in a specific culture cell medium. A method (EPISPOT) that combines the capacity of CTC to secret specific markers and grow-up in specific cell medium was developed (see Table 2).

### 7.1. Magnetic Separation

To use immune-magnetic detection system the first step is to deplete the whole blood of the red cells (by lysis buffers or density gradient) to obtain the PBMC. After, the magnetic particles coated and surrounded by a specific antibody are added to the PBMC supposing containing the CTC. Labeled cells are then collected by applying a magnetic force while nonlabeled cells are containing in the supernatant and are discerned. This use of magnetic beads to catch specifically CTC is called “positive selection” [40, 47, 49].

Since a large number of leukocytes (potential source of false negative CTC) still remain trapped with the cells, some methods include a “negative selection” of leukocytes (with anti-CD 45 beads for example) followed with a “positive selection” with antibodies specific to epithelial cells (EpCAM, CK) [47, 169, 170]. The problem of this procedure is that gets ride the majority of leukocytes but still hold in nonmalignant epithelial cells and loses tumor cells which whish do not express epithelial antigens and/or are lysed during the first step [47, 171].

The methods using antibodies like immune-magnetic methods (MACS system, Deanabeads Invitrogen, macroiron beads, ferrofluid(colloidal iron-) based systems) to enrich the sample will induce false-positive extraction [168]. For example, antibodies against cytokeratin (CK) or other epithelial-specific antigens have been reported to bind both specifically and nonspecifically to macrophages, plasma cells and nucleated hematopoietic cells precursors. The nonspecific binding of the antibodies involves Fc receptor-bearing leukocytes and monocytes or illegitimate expression of epithelial antigens in normal hematopoietic cells. Some of these positive cells are morphologically difficult to distinguish from CTC. Variable numbers of epithelial cells have been found in peripheral blood of subject without malignancy in some physiopathological conditions like benign epithelial proliferative diseases, inflammation, surgeries, and tissue trauma [47, 65, 169, 172–174]. Moreover, epithelial CTC may lose epithelial markers during dissemination through the process called epithelial-to-mesenchymal transition (EMT). Since the epithelial markers that get lost during EMT may include markers used for CTC measurement, underestimation of the actual CTC number may occur, inducing de facto false-negative results [46, 47, 49, 175, 176]. In the case that the method induce false positive, the problem can be diminished avoided using a second marker or a full panel of markers and techniques (see below) to characterize the CTC, like RT-PCR, immunocytochemistry or immunefluorescence, morphology by optical microscopy [37, 47, 49]. In another hand, in the case of the false negative results the doubt persist, and only strict followup of the patient by repeating the detection of the CTC can potentially eliminate this doubt. No available antibodies are 100% tumor or tissue specific [13, 47, 172]. To isolate CTC a method using a ligand biotinylated was used. Biotinylated asialofetuin, a ligand of asialoglycoprotein receptor, was experimented and followed by magnetic separation or density gradient (Ficoll-Paque PLUS; GE Healthcare). The cells were identified by microscopy, FISH, immunofluorescence staining, flux cytometry, and RT-PCR. This technique shows 81% specificity and 20 cells/5 mL for the sensitivity [177]. This promising approach has to be confirmed in a larger cohort of patients and still depends on the receptors expressed at the surface of the CTC.

### 7.2. Culture of CTC

After isolation of the CTC by the different methods described above, to increase the number of CTC, the primary tumor cells can be cultured in the specific culture medium [169]. The good conditions of culture growth and specially the culture medium leading the growth of the CTC, but not the other epithelial or nonepithelial cells, has to be determined through an experiment. Some companies propose commercial kits. For example, The Cancer Cell Isolation Kit from Panomics includes lysis buffer to increase the number of CTC. One of the main problems is that cultured cells can lose their original markers and derive. Mimicry of tumoral microenvironment in vitro is particularly difficult because for most tumors it is largely unknown. Another problem is that the samples containing the CTC are usually contaminated by stromal cells like fibroblasts, which create competition in the Petri dish. After few days, only the fibroblasts are present in the flask.

### 7.3. Epithelial ImmunoSPOT (EPISPOT)

A technique that allows the detection of only viable cells after a CD45^+^ cell depletion was introduced for CTC analysis from bone marrow aspirates and blood samples [40, 43, 151]. This technique was designated EPISPOT (epithelial ImmunoSPOT). It is a protein-secreting profiling based on the secretion or active release of specific marker proteins using an adaptation of the enzyme-linked immunospot technology. As immunospots are the protein fingerprint left only by the viable releasing epithelial cells, a cell culture is needed to accumulate a sufficient amount of the released marker proteins (Table 2). The dying cells do not secrete adequate amounts of protein and are not detected [40, 150–152]. This assay can also provide important information on the profile of secreted proteins potentially relevant for metastasis formation. However, this technique has still to be validated in large-scale clinical studies on cancer patients [40, 151]. After the enrichment and isolation of the CTC, the next step is to identify, characterize, and finally enumerate them. The CTC can be identified by indirect or direct methods. But these important steps need tumor markers specific to the CTC seeked.

## 8. Experimental Models

### 8.1. HCC Mouse Models

Over decades, different HCC mouse models have been developed.

Chemically induced HCC-models are diverse and not always reproducible. The chemicals usually used are diethylnitrosamine (DEN), peroxisome proliferators, aflatoxine, carbon tetrachloride (CCl_4_), choline deficient diet or thiacetamide [178, 179]. Transgenic mouse models were also developed, for example, mice that contained HBV or HCV viruses or expressed specifically oncogenes (c-myc, c-myc + E2F1) or growth factors (TGF-*α*, TGF-*α* + c-myc, EGF, FGF19, GMNT, PDGF, *α*1-antitrypsin) [178]. Circulating tumor cells were not looked for in any of these animal models. One reason is the huge differences between models and the presence of specific markers for each situation.

In order to solve these problems, researchers developed ectopic implantation that is fast and easy to perform. However, there is still many differences between the cell lines, no direct interaction with the liver tissues and difficulty to export to humans [178].

### 8.2. Orthotopic Implantation

Orthotopic implantation is a more suitable model because the cells are directly implanted in the liver tissue. Nevertheless, the procedure is challenging. There are big differences between cell lines and the choice of the markers is still limited [178]. Mechanisms leading to tumoral cells spreading are ill known. Currently, there are few models of orthotopic implantation of human tumoral cells [180, 181]. An experimental model of human orthotopic HCC transplantation in NOD/SCID (nonobese diabetic/several compromise immunodeficient) mice allows to generate and to modulate CTC [180, 181]. In this mouse model, tumoral spreading is an early event during tumoral development and the number of CTC is directly correlated to the tumor size.

When injected under the liver capsule, a primary tumor develops and continuously yields circulating tumor cells. In addition, the CTC could be modulated after tumor removal. Liver tumor removal led to a very low level of tumoral cells in blood 30 days later. After complete tumor removal, the number of CTC significantly decreases but still remains detectable even at a low level. The FACS was used to detect CTC (detection of human HLA marker in mouse bloodstream). The reality of CTC was then demonstrated. An important finding is that the bone marrow could be early and permanently colonized by CTC [181].

### 8.3. Small Imaging Animal Models

With the recent development of the small imaging apparatus (example: IVIS Lumina II XR Imaging System, positron emission tomography) to study development and the progression of diseases in the live animals like rheumatism, a new area to study CTC in live animals is open. This technique was applied to study the CTC in ectopic or orthotopic HCC cell lines implantation. As we discuss below, the lack of specific HCC markers makes CTC studies very challenging. The idea is to bind luminescence tag (luciferase, yellow fluorescence, or red fluorescence proteins) in the hepatoma cell lines injected in the liver that be detected by bioluminescence machine. For example, thymidine kinase-luciferase was placing under the transcriptional control of endogenous AFP promoter to develop a transgenic mouse model that injected with DEN will develop HCC [182]. The development of the HCC was followed in the live animal by bioluminescence and PET analyses. The inconvenient of this method is that the HCC model has to express AFP. To avoid this problem hepatoma cell lines where engineered with luciferase (HCC-LM3) [183] or red luminescence protein [184]. This approach to study CTC in the context of HCC is very promising, but the major problem is the sensitivity of the bioluminescence machine. This approach has not yet studied in the context of CTC in the blood or in organs other than liver.

In vivo flow cytometry method to detect CTC from HCC has been developed. This method combined the flow cytometry technology that can detect specific fluorochromes based on their wavelengths, the specificity of these fluorochromes attached to an antibody that detects the CTC or fluorescently labelled cells as described in Li et al. paper [185]. Briefly, fluorescence signal from a given circulating cell population is recorded as the cells pass through the slit of light. Confocal detection of the excited fluorescence enables continuous monitoring of labeled cells in the upper layers of scattering tissue, such as the skin of a mouse ear. Based on algorithm and data analysis the computer is able to estimate the number of CTC in the blood stream. This method is still used in animal models to detect CTC from HCC but as promising future to detect CTC in human patients.

## 9. Conclusion

There are two major problems to detect circulating hepatocarcinoma cells in the human blood. The first problem is the low number of specific markers known. The second problem is that few cells are present in the bloodstream. To overcome these problems, few years ago new approaches have been developed such as the techniques to study membrane proteins by mass spectrometry and the development of fluorescent hepatoma cells.

Nowadays, these procedures are not suitable in clinical practice. However, it is undeniable that early detection of tumors and metastasis is urgently needed in medicine and these new exciting techniques and findings are changing our point of view of carcinogenesis very fast. In the future, CTC detection will certainly be an important diagnostic tool in cancer patients, providing a new and accurate assessment of lesion staging.

## Figures and Tables

**Table 1:** Evaluation of serum alpha fetoprotein as a marker of circulation tumor cell in different hepatocellular carcinoma studies. nRT-PCR, nested RT-PCR; qRT-PCR, quantitative RT-PCR.

Author	PCR	Sensitivity	Cases	Samples	Positivity	Predictability of recurrence
[55]	qRT-PCR	1 CHC/10^7^	37 136	Blood BM	18% 28%	No Yes
[64]	qRT-PCR	1 CHC/10^6^	38 25	Blood BM	10% 48%	Yes No
[56]	nRT-PCR	5 cells/1 mL	24	Blood BM	29% 43%	Suspect
[54]	Competitive RT	10 cells/9 mL	22 11	Blood BM	26% 45%	NA
[90]	RT-PCR	NA	18	BM	93%	No
[61, 62]	nRT-PCR		33	Blood	52%	Extrahepatic metastases
[57]	nRT-PCR	15 cells/mL	64	Blood	36%	Extrahepatic metastases
[58]	nRT-PCR	1CHC/10^5^ mono	20	Blood	25%	No
[91]	nRT-PCR	10^−6^ *μ*g/*μ*L of RNA	33	Blood	54%	Yes
[92]	nRT-PCR	1CHC/10^5^ mono	87	Blood	36%	Yes
[93]	nRT-PCR	1 CHC/10^7^ mono	85	Blood	26–45%	No
[88]	RT-PCR	1 CHC/10^6^	52	Blood	25%	No

**Table 2:** Summary of advantages and disadvantages of the methods of CTC enrichment. CTC, circulating tumor cell; CTC-Chip, circulating tumor cell chip; EPIPSPOT, epithelial immunospot; FACS, fluorescence-activated cell sorting = flow cytometric; FAST, fiber-optic array scanning technology; ICH, immunocytochemistry; ISET, isolation by size of epithelial tumor cells; MACS, magnetic cell sorting; MEMS, microelectromechanical system.

Methods of enrichment	Advantages	Disadvantages	References
Nonspecific			

Density (OncoQuick, Ficoll, UNI-Set)	(1) Isolation of whole and living CTC, (2) using another method of enrichment more specific (immunomagnetic beads), (3) cytopathology, cytological staining, ICH, FISH, and so forth can be performed (4) RNA, DNA extractions followed by RT-PCR or PCR, respectively, can be done.	(1) Nonspecific, (2) rare CTC can be lost in the plasma fraction or trapped among erythrocytes and neutrophils, (3) low and variable sensitivity, (4) Depends on the type of CTC, temperature, and centrifuge, (5) expensive.	[47, 65, 139, 140]

Size (MEMS, ISET)	(1) Easy, (2) precise counting of the cells per mL of blood, independently of the volume of the blood treated, (3) Allowing cytopathology, cytological staining, ICH, FISH, and so forth, (4) Allowing Microdissection followed by (5) RNA, DNA extraction followed by RT-PCR or PCR respectively (6) Avoiding multiple steps, (7) Increasing sensitivity (1 single CTC can be detect from 1 mL of blood).	(1) Nonspecific, (2) CTC can go through the filter, (3) cells can be damaged, (4) expensive, but less than the immunomagnetic beads.	[47, 68, 124, 141, 142]

Cytospin	(1) Easy, (2) Allowing cytopathology, cytological staining, ICH, FISH, and so forth, (3) Allowing Microdissection followed by (4) RNA, DNA extraction follow by RT-PCR or PCR, respectively.	(1) Increasing the mortality of the CTC.	[65, 143–146]

Lysis Buffer (Qiagen)	(1) Easy, (2) the cells harvested by this method can be reenriched or analysed by the methods already described, (3) low cost.	(1) Increasing the mortality of the CTC, (2) low sensitivity.	[147–149]

Specific			

ImmunoMagnetic Beads (MACS system, CellSearch)	(1) Specific, (2) morphological analysis of CTC, cytopathology, cytological staining, ICH, FISH, and so forth, (3) multiple labelling of antigens on CTC, (4) direct quantification of CTC.	(1) Subjective analyses for CTC identification, (2) time-consuming screening of tumor cells, (3) needing specific marker(s) and antibody available, (4) expensive	[40, 150–152]

EPISPOT	(1) High sensitivity, (2) detection of viable CTC, (3) detection of secreted proteins	(1) Protein must be actively secreted, shed, or released, (2) no identification and isolation of secreting cell possible.	[40, 150–152]

FACS	(1) High sensitivity, (2) technique for counting, examining, and sorting microscopic particles (CTC) suspended, (3) simultaneous multiparametric analyses of the physical and/or chemical characteristics.	(1) Need the apparatus, (2) high cost.	[140, 153, 154]

FAST	(1) High sensitivity, (2) can detect rare events.	(1) Fluorescent dyeconjugated antibodies, (2) specificity depend on the antibodies, (3) very expensive.	[48, 89, 141, 155]

CTC-Chip	(1) High sensitivity, (2) detection of viable CTC,	(1) Detecting only cytokeratin ± CTC, (2) needing to control precisely laminar flow conditions, (3) expensive.	[40, 156]
